# Vom stadträumlichen Pionier zur Kulturstätte inmitten der Pandemie. Musikclubs als (sozial-)räumliche Akteure urbaner Musikökosysteme

**DOI:** 10.1007/s00548-023-00847-0

**Published:** 2023-05-03

**Authors:** Robin Kuchar

**Affiliations:** Institut für Soziologie und Kulturorganisation, Leuphana Universität Lüneburg, Universitätsallee 1, C5.221, 21335 Lüneburg, Deutschland

**Keywords:** Musikclub, Kulturstätte, Stadtraum, Livemusik, Covid-19, Music venue, Cultural institution, City space, Live music, COVID-19

## Abstract

Musikclubs gelten als bedeutende Experimentierräume der populären Musik, als Ort des informellen Austauschs lokaler Musikszenen und als Knotenpunkte szenebasierter Wertschöpfungsprozesse urbaner Popkultur. Ihre (sozial-)räumlichen Strategien und Ausprägungen zeigen dabei eher in Richtung eines auf Kultur orientierten Selbstverständnisses sowie auf alternative, gelebte und mit ihrem Standort verwurzelte Räume lokaler Nachtökonomien. Dieser Artikel betrachtet (sozial-)räumliche Austauschprozesse von Musikclubs, lokaler Musikszene, Stadt und Music Industries und geht der Frage nach, welche stadträumlichen und kulturellen Implikationen mit den Auswirkungen der Covid-19-Pandemie für diese im Kontext des städtischen Musikökosystems verbunden sind. Während die ökonomischen Folgen auf der Hand zu liegen scheinen, interessieren hier im Besonderen die Zuschreibungen von Musikclubs für die Stadt, der räumliche Wert von Livemusik und kulturellem Erbe sowie die Frage nach der bau- und planungsrechtlichen Aufwertung von Clubs und Livespielstätten während des Lockdowns im Frühjahr 2021.

## Abstract (en)

Music clubs are regarded as important experimental spaces for popular music, as places of informal exchange for local music scenes, and as hubs of scene-based value creation processes of urban pop culture. Their (socio-)spatial strategies and manifestations tend to point toward a culture-oriented self-understanding, as well as toward alternative, lived spaces of local night economies rooted in their location. This article looks at (socio-)spatial exchange processes of music clubs, local music scenes, city and music industries, and explores the urban-spatial and cultural implications associated with the impact of the COVID-19 pandemic for these in the context of the urban music ecosystem. Although the economic consequences seem obvious, of particular interest here are the attributions of music clubs to the city, the spatial value of live music and cultural heritage, and the question of the upgrading of clubs and live venues in terms of building and planning law during the lockdown in spring 2021.

## Einleitung

**Abb. 1:**
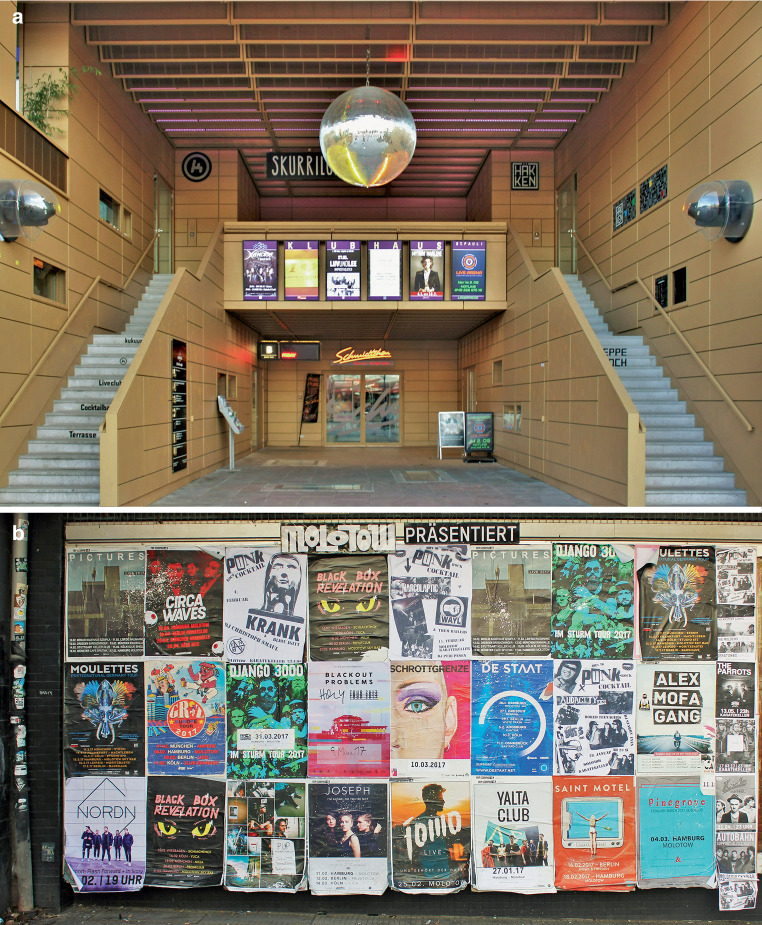
Zwei ‚Welten‘ von Clubkultur: Plakatwand des Molotow Clubs und Eingang zum Klubhaus St. Pauli. (Foto: Robin Kuchar)

Im Mai 2022 veröffentlichte das Clubkombinat Hamburg e. V. zusammen mit über 50 erstunterzeichnenden Musikclubs, Initiativen und Veranstalter*innen das Manifest *Wir brauchen Räume*, in dem die Forderung nach einer kulturintegrierten Stadtentwicklung für Hamburg dargelegt wird (Clubkombinat Hamburg e. V. 2022a). Dies erscheint im ersten Moment überraschend – denn durch verschiedene Maßnahmen und Rettungsschirme zeigt sich die Zahl dauerhafter Schließungen von Livemusikspielstätten über die Covid-19-Pandemie in der Hansestadt bislang sehr niedrig (Clubkombinat Hamburg e. V. 2022b). Bei näherer Betrachtung wird allerdings deutlich, dass nach fast zwei Jahren durchgehender Ruhe zum einen *alte* Diskurse um die von (Live‑)Musik ausgehenden „Beeinträchtigungen des Stadtraums“ (Twickel 2022, ohne Seite (o.S.)) wie etwa Lautstärkeproblematiken neu aufflammen. Zum anderen machen aktuelle Berichterstattungen und Interviews mit Clubbetreibenden, Veranstalter*innen und Musikschaffenden auf Long-Covid-Symptome des Livemusikmarktes aufmerksam, die sich vor allem für kleinere Spielstätten sowie weniger bekannte Künstler*innen und Promoter als kritisch herausstellen (Volkmann 2022; Nagel 2022; Fischer 2022; NDR 2022; Abb. 1).

In diesem Beitrag soll daher der Blick auf die stadt- und sozialräumliche Situation unabhängiger kleiner und mittlerer Musikclubs gerichtet werden. Es stellt sich die Frage, inwiefern Covid-19 die Beziehung von Livemusik, Szene und Stadtraum und damit die Bedeutung von Musikclubs als zentrale Akteure lokaler (Live‑)Musiknetzwerke (Frith 2013; Lange/Bürkner 2013) verändert hat. Hierbei soll vor allem aufgezeigt werden, wie sich der plötzliche temporäre Verlust des physischen Clubraums auf die dort stattfindenden sozialen und kulturellen Wertschöpfungsprozesse der städtischen Musik- und Nachtökonomie auswirkt und welche Langzeitfolgen hier nach aktuellem Stand zu erwarten sind. Zur Klärung dieser Fragen sollen zunächst die Funktionen des Musikclubs und seine (sozial-)räumlichen Umfelder kurz erläutert und diese in den Diskurs um musikalische Ökosysteme eingebettet werden. Im Anschluss daran erfolgt ein Blick auf die konkreten Auswirkungen der Covid-19-Pandemie. Zum Abschluss sollen die sich daraus abzeichnenden Entwicklungen unter Einbezug von Kultur- und Stadtpolitik diskutiert und mit der Berichterstattung um die gegenwärtige Situation von Musikclubs im Sommer 2022 in Verbindung gebracht werden.

## Clubraum, Stadt und musikalisches Ökosystem

Musikclubs gelten als bedeutende Experimentierräume der populären Musik, als Ort des informellen Austauschs lokaler Musikszenen und in vielen Fällen – von Cavern Club über Starclub, CBGBs bis Berghain – als Ikonen und räumliche Zentren urbaner Popkultur (Shuker 2005; Pütz 1999). Mit einem häufig auf der Umsetzung eigener kulturellen und ästhetischen Ideen basierenden Selbstverständnis sowie der Bindung an lokale Szenen und urbane Netzwerke der Musikproduktion, können sie in Relation zu städtischen Steuerungs- und Planungsprozessen als kulturelle Initiativen *von unten*, also als *Bottom-up*-Akteure im Gegensatz der *top down* orientierten Kulturpolitik und Stadtplanung verstanden werden (Drevenstedt 2020; Kuchar 2020; Kirchberg 2014).

In Kombination mit der mehr oder weniger temporären Nutzung und Umgestaltung verfügbarer Gebäude und Flächen nehmen Clubräume die Funktionen geschützter Treffpunkte und (sozial-)räumlicher Knotenpunkte für spezifische Communities und Tastecultures wahr. Dabei gelten sie, wie zum Beispiel der Golden Pudel Club in Hamburg oder Ilses Erika in Leipzig, als kollektive Freiräume, Orte des ästhetischen und sozialen Experimentierens oder als halböffentliche Wohnzimmer (Steets 2007; Vogt 2005; Currid 2007; Kronenburg 2022).

Mit dieser Ausrichtung verkörpern sie einerseits idealistischere und weniger kommerziell ausgerichtete Vorstellungen von Kultur, die sie von der Konzentration und Kommerzialisierung der globalen Livemusikindustrie mit ihren mächtigen Marktführern wie Livenation oder Eventim abgrenzen (Frith 2013; Budnick und Baron 2011). Andererseits nehmen sie im Rahmen von lokalen und translokalen Szeneökonomien die Rolle als Meinungsführer und *Tastemaker* ein und sind somit zentral an sozialen und kulturellen Wertschöpfungen lokaler Netzwerke der Musikproduktion, wie etwa dem Reputationsaufbau von Künstler*innen oder neuer Musikstile, beteiligt (Lange und Bürkner 2013, S. 157f.). Aus kulturökonomischer Sicht fungieren sie entsprechend als Bindeglied zwischen ästhetischem Experiment und Musikmarkt (Kühn 2017; Pütz 1999).

Als urbanes Phänomen ist der Musikclub ein unmittelbarer Bestandteil des städtischen Raumes – auf baulicher wie auch auf symbolischer und kultureller Ebene (Steets 2007; Kuchar 2020; Drevenstedt 2020). Der *Szenestatus* und die damit verbundenen symbolischen Zuschreibungen bilden dabei eine wichtige Grundlage für die Beziehung von Club und Stadt. Als authentisch wahrgenommene und erlebte Orte (Zukin 2010) besitzen sie nicht nur ein enormes symbolisches Potenzial zum *Placemaking*, sondern haben über ein subversives „anti-heritage“ bzw. „self-authorising music heritage“ (Roberts/Cohen 2014, S. 252f.) den Status popkulturellen Erbes erreicht (Strong 2022; Bennett und Rogers 2016). Dies ist vor allem an stilprägenden und allgemein bekannten Beispiele wie Starclub, Berghain oder Studio 54 festzumachen – aber auch für weniger bekannte Clubräume wie Molotow in Hamburg, Blue Shell in Köln oder SO36 in Berlin der Fall. Schilder und Tafeln mit Namen der hier aufgetretenen Bands wie im Hamburger Molotow zeugen von einem gegenseitigen Aufbau von Wertschätzung zwischen Club, Künstler*innen und Publikum (Lange und Bürkner 2013), der sich sowohl physisch wie auch symbolisch in diese Orte einschreibt.

Die räumliche Wahrnehmung von Musikclubs als popkulturelle Leuchttürme verweist allerdings auch auf die oft in Presse und im Rahmen stadträumlicher Konflikte beschriebenen Problematiken um die Rolle von Clubräumen als städtische Pioniere und Kulturalisierungsagenten sowie gleichzeitig als Opfer von Aufwertung- und Instrumentalisierungsprozessen, zum Beispiel der Nutzung ihres symbolischen Wertes für Stadtmarketing und Tourismus (u. a. Holm 2010, Neumann 2012; Kirchberg und Kagan 2013; Reckwitz 2012). Beispiele hierfür sind unter anderem der über mehrere Jahre anhaltende Diskurs um die 2013 durchgeführte Räumung des Molotow-Clubs und der Esso-Häuser am Spielbudenplatz in Hamburg und der Streit um die Re-Integration des Clubs in den Neubau des Palomaviertels – oder die Schließung der Griessmühle in Berlin mit der damit verbundenen Initiative „Save our Spaces“.^1^

Wie aus Forschungen zur Langzeitentwicklung von Musikclubs in Hamburg hervorgeht, sehen sich Clubräume und ihre sozialräumlichen Funktionen einer Vielzahl von Herausforderungen und Veränderungen in ihren sozialen und räumlichen Umfeldern ausgesetzt (Kuchar 2020). Die fortschreitende Kommerzialisierung von Livemusik- und Nachtökonomie und der damit steigende ökonomische Druck auf lokale Akteure (Frith 2013; Seliger 2013) spielen hier ebenso eine Rolle wie die stadträumlichen Diskurse um Verdrängung und Instrumentalisierung (u. a. Chatterton und Hollands 2003) der immer fluider werdenden Szenestrukturen (Kuchar 2023).

**Abb. 2:**
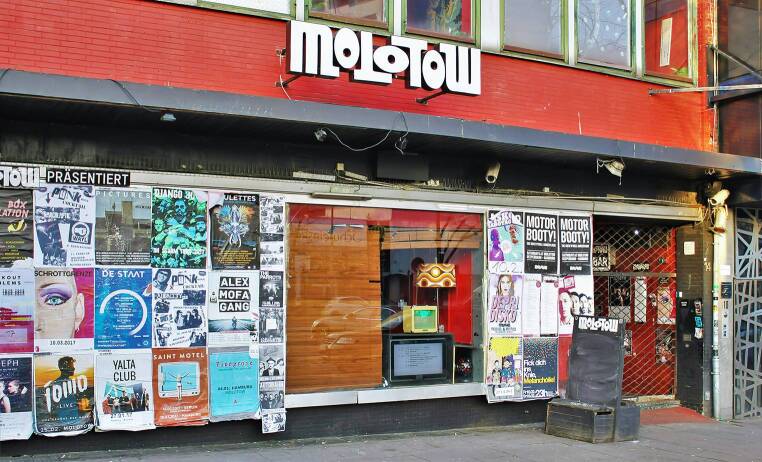
Außenansicht der Molotow-Clubs am aktuellen Standort am Nobistor. (Foto: Robin Kuchar)

Einen wichtigen Faustpfand der Clubs stellt in diesem Prozess ihre Bedeutung als städtischer Imagefaktor sowie die allgemein steigende Anerkennung von Clubräumen als kulturelle und wertschöpfende Akteure dar, die maßgeblichen zur Position von Clubs im Stadtraum beitragen (Kuchar 2020). Die lokale Kultur- und Stadtentwicklungspolitik nimmt dabei als Ansprechpartner sowie als Förderer eine immer wichtigere Rolle für lokale Livemusikszenen und damit auch für Clubs ein, was sich in der Untersuchung drei spezifischer Fälle in Hamburg zeigt: Obwohl sich die drei Clubs Mojo, Molotow (Abb. 2) und Golden Pudel zwischen Professionalisierung und Annäherung an die Musikindustrie (Mojo), Bewahrung alternativer Musikkultur (Molotow) und klarer gegenkultureller Orientierung (Golden Pudel) in unterschiedliche Richtungen entwickelt haben, hängt ihr Fortbestehen nicht mit einer großen öffentlichen Unterstützung, sondern direkt mit dem wahrgenommenen symbolischen und stadträumlichen Wert von Clubräumen seitens der Stadt zusammen (Kuchar 2020).^2^ Während der Mojo Club durch politisches Einwirken in den Neubau der Tanzenden Türme an ursprünglicher Adresse integriert wurde, wurde der Fortbestand und Wiederaufbau des Golden Pudel Club 2017 durch eine sechsstellige Fördersumme gesichert (Kuchar 2020). Im Falle des Molotow Clubs wird die Integration des Clubraums in den Neubau des Palomaviertels mit über 1,5 Mio. € gefördert (Grund 2020).

Unter Berücksichtigung dieser Zusammenhänge können stadträumliche Austauschprozesse von Clubräumen nicht isoliert von ihren räumlichen und sozialen Umfeldern, in die sie eingebettet sind, betrachtet werden. Eine hierfür in den letzten Jahren *in Mode* gekommene Perspektive ist die Betrachtungsweise des *kulturellen* oder *musikalischen Ökosystems* (u. a. Behr et al. 2016; Schippers 2016). Der Ansatz integriert in einer ganzheitlichen und integrativen Betrachtungsweise nicht nur die verschiedenen Produktionsebenen und Interessenslagen innerhalb – in diesem Fall – des lokalen Livemusiksektors, sondern „views live music … as a (inter)local network of different social actors within live music and beyond (e.g. regulators, policy makers, sponsors)“ (van der Hoeven et al. 2020, S. 28). Während die Ecology-Perspektive in kulturpolitischen und stadträumlichen Entwicklungsprozessen, zum Beispiel in Form kulturintegrierter Stadtentwicklungskonzepte, neben wirtschaftlichen vor allem auch kulturelle und soziale Wertschöpfung durch Kultur in den Mittelpunkt der Strategien und Interventionen stellt (Holden 2004, 2015; Stadt Köln 2019), liegt ihr analytisches Potenzial vor allem in der Betrachtung externer Einflussfaktoren auf kulturelle Systeme und Netzwerke (Van der Hoeven et al. 2020; Schippers 2016). Besonders für die Verbindung interner (Szenestruktur, Organisation von Wertschöpfungsprozessen im Club et cetera) und externer Faktoren (Stadtpolitik und -planung oder für die Einflüsse der Covid-19-Pandemie) auf kulturelle Felder erscheint die ökologische Perspektive als ein nützlicher Ansatz. Allerdings ist zu beachten, dass für das „Buzzword Ecology“ (Behr et al. 2016, S. 6) im kulturellen Kontext noch einige Unklarheiten bestehen und hier (noch) nicht von einem in sich theoretisch und methodologisch konsistenten Ansatz gesprochen werden kann (DeBernard et al. 2021).

Musikclubs können allerdings in jedem Fall als ein Teil des städtischen Livemusik-, oder weiter gefasst, des städtischen Musikökosystems verstanden werden. Stadtplanung, Stadtpolitik und gesamtgesellschaftliche Notlagen wie die Covid-19-Pandemie lassen sich für die Clubs als aktuell entscheidende externe Einflussfaktoren für sie und die sie umgebenden Musiknetzwerke identifizieren. Auf die konkreten sozialräumlichen Veränderungen von Clubräumen *von außen* – sprich durch Covid-19 – soll im folgenden Abschnitt näher eingegangen werden.

## Die Auswirkungen von Covid-19 für die Clublandschaft

Das plötzliche und längerfristige Erliegen des Livemusik- und Clubbetriebs in einem internationalen Kontext (Carr 2022; Anderton 2022) führte im Frühjahr 2020 nicht nur zum Verlust jeglicher Livemusikpraxis, sondern ebenso zu einer Entleerung jeglicher Räume der Live- und Nachtökonomie (Taylor et al. 2020; Assiter 2020). Im Fall der Livemusik kann dies grundsätzlich mit der temporären Auflösung ihres räumlichen Wertes als „relationship between live music and the built environment, as constituted by the dimensions of performing, (re)developing and narrating the urban space“ (Van der Hoeven und Hitters 2020: 155), aber auch als plötzliches Verschwinden der physischen Präsenz von Musikszenen und -netzwerken im Stadtraum beschrieben werden.

Neben der erwartbaren wirtschaftlichen Krise – nicht nur für Clubs, sondern der gesamten Livemusik- und Veranstaltungsbranche (Carr 2022; Krause et al. 2022; Nolte 2021) – ist hier auf Basis erster empirischer Untersuchungen zwischen den direkten Auswirkungen auf Clubs, den Folgen für ihr soziales Umfeld (Besucher*innen, Musikschaffende, Szene) und der im Club stattfindenden soziokulturellen Wertschöpfungsprozessen sowie den stadträumlichen Veränderungen zu differenzieren.

**Abb. 3:**
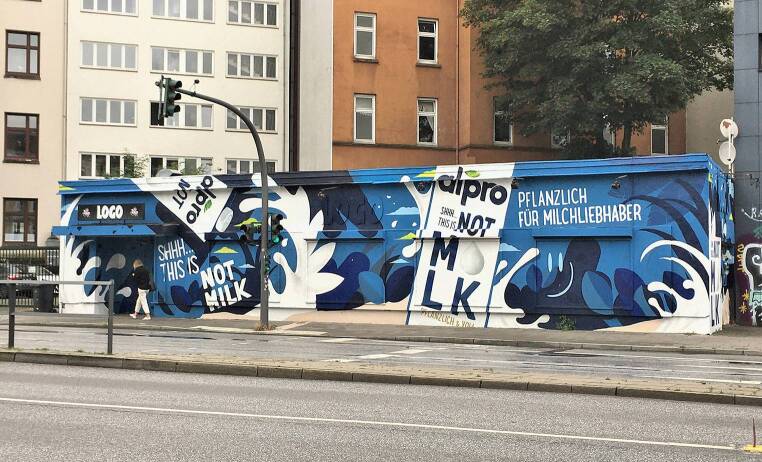
Der Hamburger Club Logo als temporäre Werbefläche für ein Milchprodukt 2021. (Foto: Robin Kuchar)

Auf Clubebene führte der Beginn des ersten Lockdowns im Frühjahr 2020 zunächst zur Ausbildung aktionistischer Strategien zum wirtschaftlichen *Überleben* wie etwa zum Verkauf von virtuellen Tickets, *Rettermerchandise* oder zur Initiierung von Crowdfunding-Kampagnen bis hin zur Umfunktionierung von Clubräumen zu Testzentren wie etwa dem Berliner KitKat Club (Kuchar et al. 2022). Dies war vor allem der Tatsache geschuldet, dass zum frühen Zeitpunkt der Pandemie noch keine Aussicht auf öffentliche Hilfen in Form von Rettungsschirmen oder dem später aufgelegten Programm Neustart-Kultur bestand (Abb. 3).

Während sich der Aspekt des ökonomischen Überlebens zumindest für die Zeit der Schließung in der Folge etwas entspannte (Krause et al. 2022), rückten Aspekte in den Vordergrund, die mit der soziokulturellen Wertschöpfung des Clubraums in Verbindung stehen: Die von vielen Clubbetreiber*innen wahrgenommene Inhaltslosigkeit ihrer Tätigkeit während der Pandemie, die sich vor allem am Fehlen einer der wichtigsten Erfolgskategorien in kulturellen Communities und -netzwerken, nämlich der sozialen Anerkennung und Wertschätzung bemisst (Currid 2007; Lange 2014), sorgte hier für eine deutliche Zunahme von Frustration und Erschöpfung. Die Abwanderung von Personal und die Dauer der pandemiebedingten Einschränkungen lassen hier zusätzlich auf die Veränderung sozialer und organisatorischer Strukturen einzelner Clubräume schließen. Clubbetreibende sprechen in diesem Zusammenhang vermehrt von „mentaler Insolvenz“ als größte Gefahr für das Überleben vieler Clubs sowie den Verlust von „Kreativität, Spontanität, … sozialer Konnektivität sowie Wagemut“ (Clubbetreiber, in Kuchar et al. 2022, S. 183).

Die Verlusterfahrung sowie der Abbruch der physisch-räumlichen Verbindung mit dem Clubraum und der musikbezogenen Community stellt auch aufseiten von Clubpublika und musikalischen Netzwerken den gravierendsten pandemiebedingten Einschnitt dar (Kuchar et al. 2022; Taylor et al. 2020; Gligorijevic 2022). Unter Musikschaffenden führten die Einschränkungen zum Zusammenbruch ihrer Auftrittsmöglichkeiten und damit zu großen Schwierigkeiten, was den eigenen Reputationsaufbau bzw. den Aufbau eigener Karrieren betrifft. Dies und die Dauer der Einschränkungen erklärt in Teilen zudem die Abkehr vieler Musiker*innen von ihren künstlerischen Tätigkeiten im Verlauf der Pandemie (u. a. Canizzo und Strong 2022). Aufseiten des Publikums und aus Sicht musikbezogener Communities und Netzwerke wird durch den Wegfall von Treffpunkten und physischen Austauschmöglichkeiten vor allem die Sorge vor einem Zerbrechen lokaler Szenen und dem Verschwinden von Clubräumen als Ort sozialer und kultureller Interaktion deutlich (Kuchar et al. 2022).

Alternative Onlineformate konnten durch ihre Einschränkungen in puncto direktem Austausch, räumlicher Umgebung und ästhetischer Erfahrung den Verlust der zentralen Bestandteile clubräumlicher Wertschöpfung (Webster et al. 2018; Behr et al. 2014; Lange und Bürkner 2013) nicht kompensieren (Vandenberg et al. 2020). Selbst zeitweilig stattfindende Veranstaltungen unter Hygienemaßnahmen wurden von Teilnehmenden trotz der Möglichkeit kulturell-ästhetischer Erfahrungen vor allem auf Ebene des sozialen Austauschs als restriktiv und reguliert wahrgenommen, was eine Analyse von Clubbesuchen des St. Gallener Clubs Palace während der Pandemie illustrativ aufzeigt (Gligorijevic 2022).

Neben einer notwendigen *Wiedergewöhnung* an soziale Nähe und räumliche Enge weisen aktuelle Untersuchungen auch auf eine generelle Veränderung des kulturellen Verhaltens über den Verlauf der Pandemie hin (Allmannritter und Tewes-Schünzel 2021). Durch die Längerfristigkeit der Einschränkungen und die damit verbundene Sensibilisierung der Risikowahrnehmung hinsichtlich einer Covid-19-Infektion kann im Clubkontext im Vergleich zur präpandemischen Situation von einer deutlichen Zurückhaltung ausgegangen werden, was die Teilnahme an Live- und Clubveranstaltungen angeht (Allmannritter und Tewes-Schünzel 2021).

Eine aktuelle Betrachtung des Livemusiksektors bestätigt diese Ergebnisse: Insbesondere im Bereich von Newcomern und musikalischen Nischen fallen viele Veranstaltungen aufgrund von zu geringer Ticketnachfrage und Unsicherheiten seitens des Publikums aus (Volkmann 2022; SWR 2022; Nagel 2022). Gleichzeitig steigt die Konkurrenz um das verbliebene Publikum. Im Livemusikbereich strahlt dies unter dem grundlegenden Kostenproblem darstellender Künste (Baumol und Bowen 1966) auch auf die Infrastruktur und damit die Clubs als Veranstaltungsorte zurück. „Für Clubs …, die zum Regelbetrieb ohne Kapazitätsbeschränkungen zurückgekehrt sind, ist das ein großes Problem: Hilfsprogramme … greifen in solchen Fällen schlichtweg nicht“ (Nagel 2022, o.S.). Die Frage nach den langfristigen Auswirkungen der Covid-19-Pandemie sowie nach den damit verbundenen Verschiebungen städtischer Musikökosysteme stellt sich für Clubräume daher aktuell mehr denn je.

## Clubräume zwischen Krise und kultureller Institutionalisierung?

Die Pandemie führte nicht nur zu einem temporären Verschwinden von Livemusik und Clubkultur aus dem Stadtraum, sondern auch zum Stillstand eines Großteils der Aktivitäten des städtischen Musikökosystems – nicht nur bezogen auf die Schließung von Veranstaltungsstätten, sondern ebenfalls auf kollektive Produktions‑, Rezeptions-, und damit auf grundlegende soziale und kulturelle Wertschöpfungsprozesse lokaler Musiknetzwerke. Im Stadtraum wurden Clubräume und mit ihnen ganze Vergnügungsviertel wie etwa die Reeperbahn in Hamburg zu „abandoned places“ – und auch nach *Rückkehr zum Regelbetrieb* zeigen sich der Livemusiksektor sowie das städtische Musikökosystem weiter in Bewegung. Wie sehen innerhalb dieser Dynamiken die Perspektiven für Musikclubs – vor allem in Bezug auf ihre Rolle im Stadtraum wie auch hinsichtlich der im Club verorteten Wertschöpfungsprozesse aus?

Die Betrachtung des Livemusiksektors weist hier deutlich auf eine Verfestigung des bereits vor der Pandemie festgestellten Auseinanderdriftens von kommerzieller Liveökonomie und lokaler Livemusikszenen hin (Frith 2013; Holt 2010). Im derzeitigen Überangebot an Veranstaltungen ziehen vor allem die Konzerte der Stars und von bekannten Künstler*innen den *Hunger* des Publikums nach dem unmittelbaren Liveerlebnis auf sich (Hartmann 2022; Volkmann 2022), während lokale und nachwuchsorientierte Veranstaltungen *zu kämpfen* haben. Hierüber jedoch einen Bedeutungs- oder Relevanzverlust von Clubräumen abzuleiten, wäre zu kurzsichtig, da dies nur eine musik- bzw. kulturökonomische Sichtweise implizieren würde.

Ganz im Gegenteil dazu ist zu konstatieren, dass Clubs in ihrem Verhältnis zur Stadt sowie zur Stadtgesellschaft über die Covid-19- Pandemie eher an Bedeutung gewonnen haben. Bemerkenswert ist in diesem Zusammenhang die Tatsache, dass gerade in der Zeit der pandemiebedingten Schließung die Stadtgesellschaft als auch die lokale Politik zu entscheidenden Faktoren für die Clublandschaft wurden. Dies betrifft zum einen die große Unterstützung der Clubräume durch die Zivilgesellschaft – vom solidarischen Verzicht auf die Rückerstattung bereits erworbener Eintrittskarten bis hin zu regelmäßigen ideellen sowie finanziellen Hilfen (Kuchar et al. 2022). Auch der in Interviews und Berichten geäußerte „hohe Vermissungsgrad“ (Gilbert 2020, S. 13) von Konzerten und Veranstaltungen während der Pandemie als auch die insgesamt sehr hohe Befürwortung öffentlicher Unterstützungsmaßnahmen für Kultur zeugen von einem hohen Maß an gesellschaftlicher Solidarität (Allmannritter und Tewes-Schünzel 2021).

Zum anderen hätte ein Großteil der Clublandschaft die Krise nicht ohne die zahlreichen Rettungsschirme und Neustart-Fonds überstanden (Initiative Musik 2021; Krause et al. 2022). Die sich intensivierende Debatte über die teils widersprüchlichen Maßnahmen und Öffnungsperspektiven (u. a. Rozbicka et al. 2021; Deutschlandfunk Kultur 2021) befeuerten zudem den politischen Diskurs um den Status der nun – ähnlich zu den traditionellen Kulturinstitutionen – fast vollständig von öffentlichen Zuwendungen abhängigen Popmusik-Spielstätten. Im Mai 2021 gipfelte die Diskussion im Bundestag mit in einem stattgegebenen Entschließungsantrag zur bau- und planungsrechtlichen Anerkennung von Musikclubs „mit nachweisbarem kulturellem Bezug“ als „Anlagen für kulturelle Zwecke“ (Deutscher Bundestag 2021, S. 2).

Dieser stellt in jedem Fall eine stadträumliche Aufwertung von Clubräumen sowie gleichzeitig eine Annäherung an den öffentlichen Kultursektor – im Gegensatz zur privatwirtschaftlichen Livemusikindustrie – dar. Die Verbindungen von Musikclubs und Politik bzw. die vonseiten der Politik bestehende Wertschätzung von Clubräumen haben sich somit nicht nur – trotz oder gerade – in einer Zeit des fast vollständigen Fehlens von Konzerten und Clubveranstaltungen intensiviert. Zusätzlich wurde über die Kulturpolitik hinaus für eine grundlegende Statuserhöhung von Clubräumen in einem für die Livemusik bedeutenden Ressort gesorgt – der Stadtentwicklung und Stadtplanung (Van der Hoeven und Hitters 2020, S. 160).

Formell und ideell scheint die Rolle von Musikclubs somit – zumindest auf dem Papier – gefestigt. Die Ursache für die aktuellen Forderungen und Warnungen seitens der Clubakteur*innen liegen vor allem in der stockenden Umsetzung des Bundestagesbeschlusses zur planungsrechtlichen Anerkennung und baurechtlichen Einstufung von Clubs als Anlagen für kulturelle Zwecke begründet (LiveKomm e. V. 2022; Backstage Pro 2021). Entsprechend gelten Clubräume in vielen Städten noch immer als Vergnügungsstätten, was aus bau- und stadtplanerischer Sicht eine deutlich strengere Regulierung (Schmid 2010), vor allen Dingen jedoch weder zusätzliche Handlungsspielräume im Falle von Konflikten oder Mietstreitigkeiten noch eine, wie im Beschluss attestierte, generelle Förderfähigkeit ,bedeutet. Viele Musikclubs sehen sich derzeit mit einer prekären Lage bezüglich ihrer wirtschaftlichen Situation, einem Schwebezustand hinsichtlich ihres bau- und planungsrechtlichen Status sowie großer Unsicherheiten, was die Fortführung von öffentlichen Hilfsprogrammen betrifft, konfrontiert. Mögliche Folgen reichen von einem wirtschaftlichen Kollaps durch die Langzeitfolgen der Pandemie über die Kündigung von Mietverträgen aufgrund finanzieller Lücken nach Auslaufen der Covid-19-Rettungsschirme bis hin zur Einschränkung des Betriebs durch strengere Auflagen.

## Fazit

Während derzeit unklar ist, wie lange die pandemischen Nachwirkungen für den Livemusikbereich noch spürbar sein werden, ist für das städtische Musikökosystem und darin vor allem für lokale Livemusikakteure von einer weiter andauernden Störung auszugehen. Die flächendeckende Umsetzung des Bundestagsbeschluss zum Status von Musikclubs könnte hier stabilisierende Wirkung entfalten, auch wenn die Notwendigkeit weiterer (kultur-)politischer Unterstützung nicht auszuschließen ist. Für Musikclubs als *benachteiligte kulturelle Räume* wäre dies auf jeden Fall ein wichtiger Schritt, um sie auch auf lokaler Ebene formell dem eigenen Selbstverständnis und ihrer kulturellen sowie sozialräumlichen Rolle anzunähern. Vor dem Hintergrund der vollständigen Wiederherstellung der für den Musikmarkt und die Kulturökonomie wichtigen sozialen und kulturellen Wertschöpfungsprozesse im Clubraum erscheint eine weitere Unterstützung zudem sinnvoll. Wie die aktuellen Ergebnisse deutlich aufzeigen, benötigen die Rekonstitution musikalischer Szenen und Netzwerke sowie der Rückgewinn und Aufbau neuer Publika Zeit – was dafür außerdem benötigt wird, sind die für diese Art von sozialem und kulturellem Austausch notwendigen Räume.

## Footnotes

1 S. hierzu u. a.: https://www.clubcommission.de/saveourspaces-petition-zur-rettung-der-griessmuehle/ (29.03.2021).

2 Eine ausführliche Analyse der Entwicklung der drei Clubs und ihrer Austauschprozesse mit Szene, Stadt und Music Industries findet sich in drei Fallstudien in Kuchar (2020).
